# Soil aggregation and aggregating agents as affected by long term contrasting management of an Anthrosol

**DOI:** 10.1038/srep39107

**Published:** 2016-12-13

**Authors:** Shulan Zhang, Renjie Wang, Xueyun Yang, Benhua Sun, Qinghui Li

**Affiliations:** 1 State Key Laboratory of Soil Erosion and Dryland Farming on the Loess Plateau, Northwest A & F University, Yangling, 712100, Shaanxi, China; 2 Key Laboratory of Plant Nutrition and the Agric-environment in Northwest China, Ministry of Agriculture, College of Natural Resources and Environment, Northwest A&F University, Yangling, Shannxi, 712100, China; 3 Shaanxi Soil and Fertilizer Station, Xian, 710000, China

## Abstract

Soil aggregation was studied in a 21-year experiment conducted on an Anthrosol. The soil management regimes consisted of cropland abandonment, bare fallow without vegetation and cropping system. The cropping system was combined with the following nutrient management treatments: control (CONTROL, no nutrient input); nitrogen, phosphorus and potassium (NPK); straw plus NPK (SNPK); and manure (M) plus NPK (MNPK). Compared with the CONTROL treatment, the abandonment treatment significantly increased the formation of large soil macroaggregates (>2 mm) and consequently improved the stability of aggregates in the surface soil layer due to enhancement of hyphal length and of soil organic matter content. However, in response to long-term bare fallow treatment aggregate stability was low, as were the levels of aggregating agents. Long term fertilization significantly redistributed macroaggregates; this could be mainly ascribed to soil organic matter contributing to the formation of 0.5–2 mm classes of aggregates and a decrease in the formation of the >2 mm class of aggregates, especially in the MNPK treatment. Overall, hyphae represented a major aggregating agent in both of the systems tested, while soil organic compounds played significantly different roles in stabilizing aggregates in Anthrosol when the cropping system and the soil management regimes were compared.

Soil structure influences soil water movement and retention, erosion, crusting, nutrient recycling, root penetration and crop yield^1^. Soil aggregates are one component of soil structure, and aggregate stability is used as an indicator of soil structure^2^,^3^,^4^. Favorable soil structure and high aggregate stability are important in improving soil fertility, increasing agronomic productivity, enhancing porosity and decreasing erodibility. Soil aggregation results from the interaction of many factors including the environment, soil management, plant influences and soil properties^5^. For a given soil, soil aggregation can be directly altered by means of management strategies that disturb the soil, including land management^3^,^6^, tillage practices^7^ or fertilization^2^,^3^,^8^, which can all impact on biotic and abiotic cementing agents^9^. In order to achieve favorable soil structures it is therefore imperative to gain an understanding of how management practices affect aggregating agents.

Abandonment of arable land has been reported as one of the most important changes in land use globally in the past few decades^10^. This approach represents one way of restoring soil conditions such as soil aggregation, by reducing soil disturbance while increasing soil organic matter in the form of biological factors that include roots, fungal hyphae, and by-products of microbial synthesis and decay^10^,^11^,^12^. In contrast, leaving land in a bare fallow form over a long period results in deteriorating soil conditions, since the lack of plants diminishes the population of arbuscular mycorrhizal fungi^13^, results in the loss of initial SOC^14^ and decreases the amounts of both dry and wet macroaggregates (>0.2 mm)^15^. Over long time periods this decreases aggregate stability^3^,^16^, whereas in the short term (e.g. five years) the effects of bare fallow treatment on aggregate stability^2^ and on structure forming biota like geophagous earthworms^17^ may be small.

Under arable cropping, fertilization, as a key management practice, has complex effects on soil aggregation^1^. A number of studies have shown that long-term application of chemical fertilizers increases the amount of macro-aggregates^18^,^19^, a process driven by improved soil aggregation due to increased SOC and biological activity^20^. By contrast, other studies have reported that the application of chemical fertilizers does not significantly affect the amount of macro-aggregates and mean weight diameter (MWD), despite increases in SOC^3^,^4^,^21^,^22^. Organic manure application can promote macro-aggregate formation^8^,^23^, but only in some instances^3^,^4^,^21^,^24^ because it can also result in an increase in the dispersion of large macroaggregates^1^. The discrepancies found among studies on soil management or fertilization practices may be related to complex effects influenced by the duration of management practices, climate and soil properties. Thus, to understand the mechanisms by which soil management affects aggregation in a given soil under specific climatic conditions, further work is essential. In this paper, we analyzed how cropland abandonment or keeping land as bare fallow affected soil aggregation and aggregating agents in comparison with continuous cropping without nutrient input, and how fertilization practices affected these aspects under cropping, in Anthrosol.

## Results

### Changes in soil properties after 21 years

Soil management regimes significantly affected SOC concentrations in soil at depths of 0–10 cm and 10–20 cm, but not at a depth of 20–30 cm (Table 1). The Abandon regime increased SOC concentration by 71% relative to the CONTROL, while the Fallow showed an SOC concentration comparable to that of the CONTROL at 0–10 cm. At 10–20 cm, the CONTROL had markedly less SOC than the other two treatments. Under Cropping, manure addition significantly increased SOC concentrations at all three depths compared to the CONTROL treatment. Application of NPK or SNPK only enhanced SOC concentration significantly in soil at a depth of 0–10 cm depth. The effects on TN concentrations of soil management or fertilization under Cropping were similar to the effects on SOC, except in subsurface layers where CONTROL showed a higher TN concentration than Fallow and Abandon (Table 1).

Soil Olsen P and available K were also significantly affected by soil management regimes and by fertilization under Cropping (Table 1). Abandon and Fallow had significantly higher Olsen P concentrations than CONTROL at depths of 0–10 cm and 20–30, while similar values were observed at 10–20 cm. Conversely, soil available K was significantly greater under Abandon than under Fallow and CONTROL for all three layers. Under Cropping, organic manure amendment greatly enhzanced Olsen P and available K concentrations at all soil depths compared with other treatments. Application of NPK or SNPK also increased Olsen P at all soil depths, and available K at depths of 0–10 cm and 10–20 cm, relative to the CONTROL levels.

Compared with continuous cultivation (CONTROL), 21 years of the bare fallow regime or cropland abandonment had no significant effect on soil pH at any depth (Table 1). Under Cropping, however, soil pH at a depth of 0–10 cm decreased considerably after 21 years of input of organic manure or straw incorporation. The EC values at 0–10 cm depth were also influenced by soil management regimes; Abandon showed a markedly higher value than CONTROL (Table 1). Application of NPK, SNPK and MNPK augmented EC values at all soil depths, with the highest values occurring under MNPK.

The clay content did not change at any soil depth after 21 years of cropland abandonment or bare fallow compared with CONTROL (Table 1). Under Cropping, fertilizer treatments did not affect soil clay content relative to the CONTROL, except in the case of SNPK application, which resulted in significantly less clay than NPK and MNPK treatments at 10–20 cm depth (Table 1).

### Soil aggregate size distribution and stability

Long term abandonment of cropland significantly increased the proportion of >2 mm macroaggregates (*P* < 0.05) and decreased the proportion of <0.5 mm aggregates (*P* < 0.05) at a soil depth of 0–10 cm (Table 2). Abandonment had no effects on the size distribution of aggregates at 10–20 cm, and only markedly reduced the proportion of 0.053–0.25 mm microaggregates (*P* < 0.05) at 20–30 cm compared with CONTROL. However, bare fallow reduced the proportions of >2 mm macroaggregates (*P* < 0.05) at all soil depths tested, and significantly augmented the proportion of <0.5 mm aggregates (*P* < 0.05) at a depth of 10–30 cm relative to CONTROL (Table 2). Thus, 21 years of bare fallow or cropland abandonment regime significantly affected MWD values compared with CONTROL at all soil depths, with a significantly higher value (*P* < 0.05) under Abandon than under the other two treatments at 0–10 cm. Fallow had significantly lower values (*P* < 0.05) than the other two treatments at 10–20 cm and 20–30 cm depths (Table 2).

Under Cropping, MNPK application significantly decreased the proportions of >2 mm macroaggregates (*P* < 0.05) at all soil depths tested and increased the proportions of 1–2 mm macroaggregates (*P* < 0.05) at 0–10 and 10–20 cm depths and the proportions of 0.5–0.25 mm macroaggregates (*P* < 0.05) at 10–20 and 20–30 cm depths relative to CONTROL (Table 2). Application of SNPK significantly decreased the proportions of >2 mm macroaggregates (*P* < 0.05) at 20–30 cm soil depth and the proportions of 0.25–0.053 mm microaggregates (*P* < 0.05) at 0–10 and 10–20 cm depths. There were increases in the proportions of 2–0.5 mm macroaggregates (*P* < 0.05) at 0–10 cm depth and the proportions of 0.5–0.25 mm macroaggregates (*P* < 0.05) at 20–30 cm depth. Application of NPK only significantly (*P* < 0.05) affected the size distribution of aggregates at 0–10 cm depth; it had little effect at depths >10 cm (Table 2). Hence, fertilization treatments did not significantly alter MWD values compared with CONTROL at any soil depth (Table 2); they only modified the size distribution of macroaggregates, especially under MNPK (*P* < 0.05). Nevertheless, across all soil depths the NPK and SNPK treatments gave the same MWD values as the CONTROL, while the MNPK treatment showed significantly lower MWD value than that for CONTROL (Table 2).

### Soil organic aggregating agents

**Figure 1:**
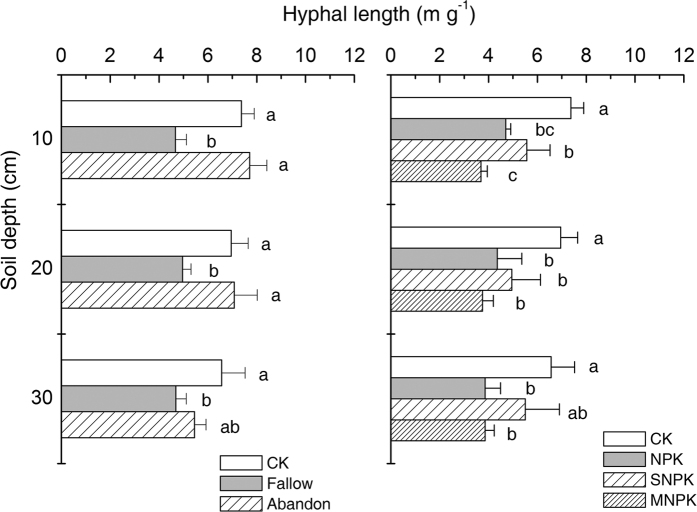
Hyphal length under contrasting soil management regimes (left panel) and fertilizer treatments (right panel). Error bars denote standard deviations (n = 3). Different small letters mean significant difference between treatments at a given soil depth at 0.05 (n = 3). Two-way ANOVA showed considerable effect of soil management regime (*P* = 0.000) and soil depth (*P* = 0.010), but no significant interaction between them (*P* = 0.077), on hyphal length, and the effect of fertilizer treatment (*P* = 0.000) and soil depth (*P* = 0.455) and their interaction (*P* = 0.846), respectively.

Hyphal length was shorter (*P* < 0.05) under Fallow than under CONTROL or Abandon at all sampling depths (Fig. 1). Long term fertilization also decreased soil hyphal length (*P* < 0.05) compared with CONTROL, except in the case of SNPK, which showed similar hyphal length to CONTROL at a soil depth of 20–30 cm (Fig. 1).

**Figure 2:**
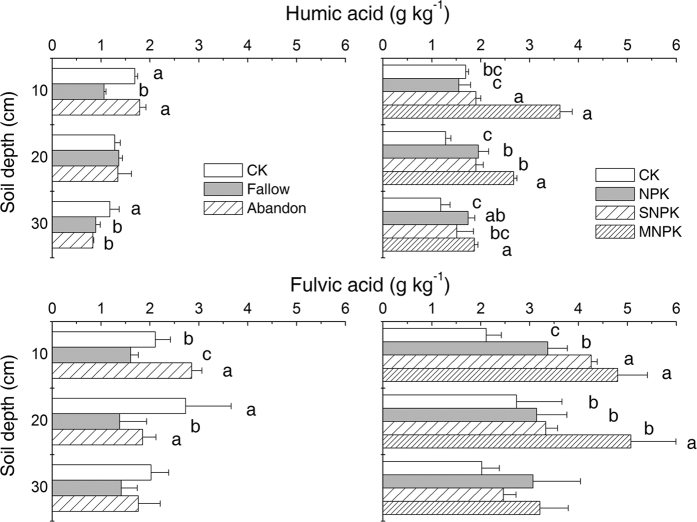
Humic (top panels) and fulvic (bottom panels) acid concentrations in bulk soils under contrasting soil management regimes (left panel) and fertilizer treatments (right panel). Error bars denote standard deviations (n = 3). Different small letters mean significant difference between treatments, at a given soil depth, at 0.05 (n = 3); no letters means no significant difference. Two-way ANOVA showed significant effects of soil management regime (*P* = 0.000) and soil depth (*P* = 0.000) and their interaction (*P* = 0.000) on humic acid content, and of fertilizer treatment (*P* = 0.000) and soil depth (*P* = 0.000) and their interaction (*P* = 0.000) on humic acid content. Two-way ANOVA showed significant effects of soil management regime (*P* = 0.001) and soil depth (*P* = 0.000) and their interaction (*P* = 0.004) on fulvic acid content, and significant effects of fertilizer treatment (*P* = 0.000) and soil depth (*P* = 0.001), but no marked interaction between them (*P* = 0.052), on fulvic acid content.

Humic acid concentrations were not altered by bare fallow or cropland abandonment regimes relative to CONTROL at 0–10 cm depth (Fig. 2). At 20–30 cm depth, however, CONTROL had much more humic acid (*P* < 0.05) than the other treatments. Under Cropping, long term addition of manure significantly increased soil humic acid concentrations (*P* < 0.05) in all layers compared with all other treatments except NPK at 20–30 cm soil depth (Fig. 2). Humic acid concentrations were also greater at 10–20 cm for SNPK (*P* < 0.05) and at 10–20 and 20–30 cm depths (*P* < 0.05) in response to application of NPK, relative to CONTROL.

Fulvic acid concentrations were significantly different (*P* < 0.05) between treatments at 0–10 cm depth, with the highest concentrations under Abandon and the lowest under Fallow (Fig. 2). At 10–20 cm, fulvic acid concentration was significantly lower (*P* < 0.05) under Fallow than under Abandon and CONTROL, and the latter two treatments gave the same values. At 20–30 cm, the three treatments showed the same concentrations. Under Cropping, fertilized treatments significantly increased soil fulvic acid concentrations (*P* < 0.05) at 0–10 cm depth compared with the CONTROL (Fig. 2), and values in MNPK and SNPK were similar and were considerably higher than in CONTROL. The fulvic acid concentration was also greater at 10–20 cm for MNPK (*P* < 0.05), but at 20–30 cm depth this treatment effect disappeared.

**Figure 3:**
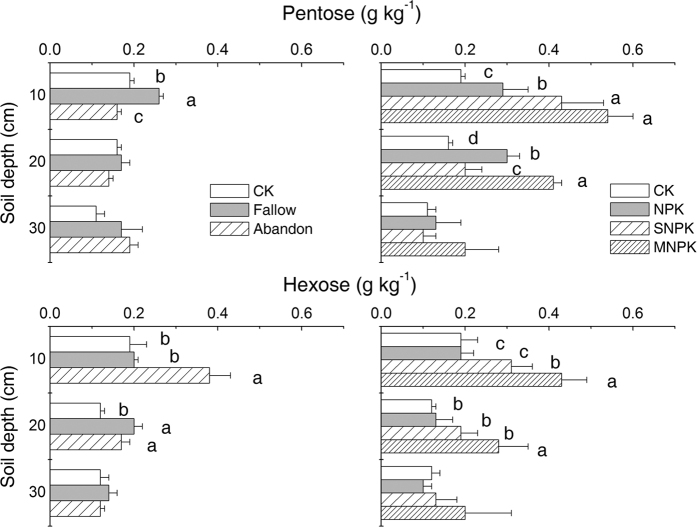
Pentose (top panels) and hexose (bottom panels) concentrations in bulk soils under contrasting soil management regimes (left panel) and fertilizer treatments (right panel). Error bars denote standard deviations (n = 3). Different small letters mean significant differences between treatments, at a given soil depth, at 0.05 (n = 3); no letters mean no significant difference. Two-way ANOVA tests indicated the effects of soil management regimes (*P* = 0.000) and soil depth (*P* = 0.000) and their interaction (*P* = 0.000) on pentose content, and the effects of fertilizer treatment (*P* = 0.011) and soil depth (*P* = 0.008) and their interaction (*P* = 0.020) on pentose content. Two-way ANOVA tests indicated the effects of soil management regimes (*P* = 0.000) and soil depth (*P* = 0.000) and their interaction (*P* = 0.000) on hexose content, and the effects of fertilizer treatment (*P* = 0.016) and soil depth (*P* = 0.006) and their interaction (*P* = 0.155) on hexose content, respectively.

Pentose concentrations were only affected (*P* < 0.05) by bare fallow or cropland abandonment relative to CONTROL at 0–10 cm depth (Fig. 3), with the highest value being found in Fallow and the lowest in Abandon. Under Cropping, fertilizer treatments significantly increased pentose concentrations (*P* < 0.05) relative to CONTROL at 0–10 cm depth, with the highest concentrations in MNPK and the lowest in CONTROL (Fig. 3).

Hexose concentrations increased in Abandon at 0–10 cm depth (*P* < 0.05) and in Fallow at 10–20 cm depth (*P* < 0.05) relative to CONTROL (Fig. 3). Under Cropping, long term addition of manure significantly increased hexose concentrations (*P* < 0.05) at 0–10 cm depth compared with other treatments (Fig. 3). The application of NPK or SNPK had a major impact on hexose concentration (*P* < 0.05) at 0–10 cm depth.

### Soil inorganic aggregating agents

**Figure 4:**
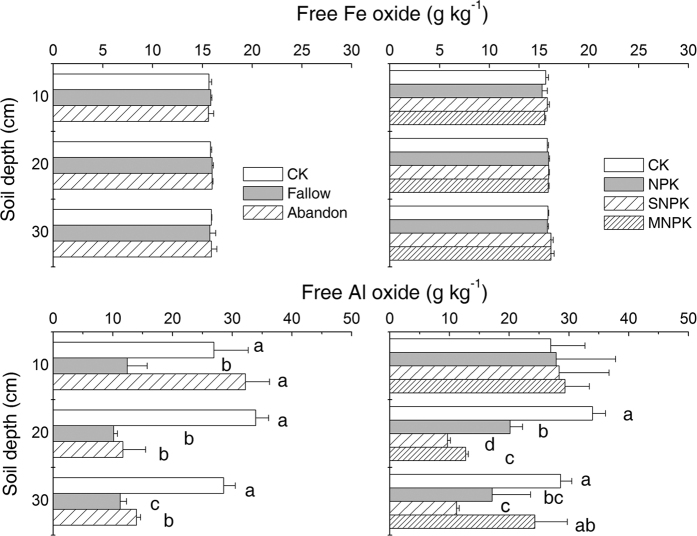
Free Fe oxide (top panels) and free Al oxide (bottom panels) concentrations in bulk soils under contrasting soil management regimes (left panel) and fertilizer treatments (right panel). Error bars denote standard deviations (n = 3). Different small letters mean significant differences between treatments, at a given soil depth, at 0.05 (n = 3); no letters means no significant difference. Two-way ANOVA tests indicate the effects of soil management regimes and soil depth and their interaction on free Fe oxide content were not significant, and the effects of fertilizer treatment and soil depth (*P* = 0.005) and their interaction (*P* = 0.337) on free Fe oxide content. They also indicate the effects of soil management regime (*P* = 0.000) and soil depth (*P* = 0.001) and their interaction (*P* = 0.000) on free Al oxide content, and the effects of fertilizer treatment (*P* = 0.000) and soil depth (*P* = 0.000) and their interaction (*P* = 0.002) on free Al oxide content.

Long term cropland abandonment or bare fallow regime did not alter free Fe oxide concentrations relative to CONTROL in any soil layer (Fig. 4). Under Cropping, organic amendments or application of inorganic fertilizers did not affect free Fe oxide concentrations relative to CONTROL in any of the soil layers (Fig. 4).

In comparison to the CONTROL, cropland abandonment resulted in markedly lower free Al oxide concentrations (*P* < 0.05) at 10–20 and 20–30 cm depths, and bare fallow had lower free Al oxide concentrations (*P* < 0.05) at all soil depths (Fig. 4). Under Cropping, no fertilizer treatments affected free Al oxide concentrations in the surface soil layer, but there were significantly lower values (*P* < 0.05) at 10–20 cm and at 20–30 cm in NPK and SNPK treatments (Fig. 4).

The CaCO_3_ concentrations were not markedly influenced by long term cropland abandonment or bare fallow regimes relative to CONTROL (Table 1). Under Cropping there were also no significant differences between fertilization treatments (Table 1).

### Relationships between aggregate stability and soil properties and aggregating agents

Pearson correlation analysis showed that MWD was significantly and positively correlated with hyphal length, humic acid, fulvic acid, free Al oxides, SOC, TN, available K and EC under contrasting soil management regimes (*P* < 0.05) (Table 3). Similarly, the proportion of >2 mm macroaggregates was strongly correlated with all of the above parameters except for EC. The proportion of 1–2 mm macroaggregates was not correlated with any of the variables listed above. Additionally, proportions of aggregates <1 mm were markedly and negatively correlated with hyphal density, fulvic acid, free Al oxides (except for the 0.25–0.053 mm class), SOC, and TN (except for the 0.25–0.5 mm class). The proportion of 0.25–0.053 mm aggregates was positively related to soil pH and negatively related to available K and EC values (Table 3).

Under Cropping, MWD and the proportion of >2 mm macroaggregates were significantly and positively correlated with hyphal density (*P* < 0.05) (Table 4). Proportions of 2–1 and 1–0.5 mm macroaggregates were related to different components of the organic matter (e.g. polysaccharide, humic and/or fulvic acid, SOC and TN concentrations) and nutrient level (P and K) and EC values (*P* < 0.05). However, the proportions of 0.25–0.5 mm macroaggregates were not related to any components of the soil organic matter or other soil properties, with the exception of hyphal density. The CaCO_3_ concentration and the pH were positively correlated with the proportion of microaggregates (*P* < 0.05) (Table 4).

## Discussion

Our results showed that cropland abandonment significantly increased the proportion of soil macroaggregates (>0.25 mm) and decreased microaggregates (<0.25 mm) at a depth of 0–10 cm but not in deeper layers. This change in aggregate distribution resulted in greater MWD (Table 2). This is in agreement with the general consensus that reducing soil disturbance or increasing the input of organic materials increases the abundance of soil macroaggregates and reduces that of microaggregates, thereby improving the aggregate stability of the soil^1^,^12^. However, bare fallow treatment for 21 years markedly affected the distribution of aggregate size and lowered aggregate stability, thus soil aggregation deteriorated at all soil depths tested due to losses of aggregating agents, such as hyphae density, soil organic matter (Table 1 and Figs 1, 2, 3 and 4) and potentially microrganisms^25^. Our results may imply that direct exposure of soil to water and heat without any cover has profound effects on soil structure, not only at the surface but also deep in the soil profile, after more than 20 years.

Under Cropping, repeated application of NPK fertilizers significantly reduced the proportion of microaggregates (<0.25 mm), though only at a depth of 0–10 cm, but it did not affect MWD (Table 2). Bandyopadhyay *et al*.^26^ also reported that long term application of NPK only resulted in a decrease in microaggregates, maintaining a similar MWD as compared with control treatment at a soil depth of 0–15 cm, but that it had no effects at 15–30 and 30–45 cm depths in an Inceptisol. However, on a Red Soil (Ultisols) with low pH (<4.0), long term application of NPK fertilizers reduced MWD^27^. In addition, the effect of straw incorporation on soil aggregation may be related to the initial SOC content. In our case, 21 years of straw return had almost the same effect as NPK on the size distribution and stability of soil aggregates, although it significantly changed some soil chemical properties (Tables 1 and 2). On a sandy loam with an initial SOC content of 4.8 g kg^−1^, increases in soil aggregation were observed after 7 years of incorporation of rice straw^28^. Long term manure amendment did not significantly affect the abundance of soil macroaggregates (>0.25 mm), microaggregates (<0.25 mm), or MWD at any soil depth (Table 2). However, across the three depths, the MNPK treated soil showed significantly lower MWD values than the CONTROL soil. Previously, Xie *et al*.^4^ reported that application of a high level of manure combined with mineral fertilizers reduced the proportions of >2 mm aggregates and increased the abundance of 0.25–2 mm aggregates; it did not affect aggregates of 0.053–0.25 mm and thus did not alter MWD. However, other studies found that manure application markedly decreased the abundance of microaggregates^26^,^29^ and improved aggregate stability^26^,^27^,^28^. These differences in results are possibly related to the ways in which soil properties and manure quantity and quality modify the effect of manure addition on aggregating agents. In addition, the relationship between aggregate stability and rates of organic input is not clear from the literature due to variations in factors such as the quality, quantity and timing of organic matter addition^30^.

Soil aggregation results from the rearrangement, flocculation and cementation of particles. It is mediated by SOC, biota, ionic bridging, clay and carbonates^1^. Our results showed that aggregate stability and the proportion of large macroaggregates (>2 mm) were strongly dependent on hyphal length and fulvic acid concentration under systems receiving no exogenous nutrient input (Table 3). This positive effect of hyphal length on aggregation is well known^31^,^32^,^33^, and may be due to substances released by hyphae, including glomalin-related soil protein^31^,^34^. Additionally, our results demonstrated that fulvic acid played a major role in the formation of large macroaggregates and increased aggregate stability, in contrast to carbohydrates such as pentose and hexose (Table 3). Martin *et al*.^35^ reported a significant and positive relationship between soil pentose and MWD in a Ferralsol. Inconsistencies in the literature regarding the role of polysaccharides in binding water stable aggregates have been reported^36^. We also found a positive effect of SOC concentration on MWD, as Jastrow *et al*.^32^ documented, but Chaudhary *et al*.^31^ found there was no effect of organic matter in their semi-arid ecosystem. These differences may be due to the different methods used for measuring the organic C and organic matter composition of soils, and to ecosystem type.

Unlike soil management regimes, under the cropping system hyphal density was the only bonding agent affecting aggregate stability and the proportion of large macroaggregates (>2 mm) (Table 4). Fertilization regimes decreased aggregate stability to varying extents, ranging from slight (NPK and SNPK) to significant (MNPK). This was probably because fertilization generally increases the levels of soil nutrients such as nitrogen and phosphorus (Table 1) and promotes plant growth, but it often reduces colonization of plant roots by symbiotic arbuscular mycorrhizal fungi (AMF)^37^, as shown here by significantly lower hyphal lengths (Fig. 1), and thus reduces the production of glomalin-related soil protein^4^, although fertilization significantly increased other organic aggregating agents such as organic matter compounds. In addition, all organic matter compounds showed a negative correlation with MWD and with the proportion of macroaggregates bigger than 2 mm, which was in contrast with the above system without any external input (Tables 3 and 4). This discrepancy in the functions of organic matter may be related to the input of electrolytes that follows manure addition, as shown by the very significantly high EC values (Table 1), which had an adverse effect on macroaggregate formation^38^. This further reflects the fact that the complex interactions of aggregating agents can be synergistic or disruptive to aggregation^1^. Organic matter compounds such as polysaccharides and humic compounds and total SOC and TN acted as aggregating agents, contributing to the formation of medium-sized aggregates (0.5–2 mm) under our cropping system (Table 4). Virto *et al*.^39^ found that an increase in the ratio of SOC to soil inorganic C (SIC) could increase the part played by SOM in aggregation in carbonate-rich soils (15–30%). Our soil has about 9% CaCO_3_; all classes of aggregates showed a higher ratio of SOC to SIC under MNPK treatment compared with CONTROL treatment, but the 0.5–2 mm group had the highest ratio^40^. This may explain the role of soil organic compounds in facilitating the formation of medium-sized aggregates. In the case of 0.25–0.5 mm aggregates, hyphal density did not benefit their formation and none of the other bonding agents significantly affected the formation of this class of aggregates, which might imply that they all have similar functions. High CaCO_3_ concentration and high pH promoted the formation of micro-aggregates (Table 4). The role of carbonates, as a source of Ca, in promoting mineral bonds and mineral-SOM interactions mediated by cation bridges has been described as being responsible for microaggregate formation and stability in several studies^41^,^42^,^43^. Overall, the part played by aggregating agents in driving aggregation was more complex under cropping than in the soil management regimes discussed above.

In summary, we conclude that on Anthrosol hyphae were an important aggregating agent and played a major role in improving aggregate stability, while soil organic compounds played significantly different functions in stabilizing aggregates in the cropping system compared with the system without any exogenous nutrient input.

## Methods

### Study site and experimental design

A long-term experiment was established in October 1990 at the Chinese National Soil Fertility and Fertilizer Efficiency Monitoring Base for Loessial Soil (N 34°17′51′′, E 108°00′48′′, with an altitude of 524.7 m a.s.l.), which is located on level land near Yangling, Shaanxi, China. The soil at the site is a silt clay loam (clay 32%, silt 52% and sand 16%), derived from loess materials, and classified as an Anthrosol (WRB 2014). At the time of establishment the topsoil (0–20 cm) at the site contained 7.44 g kg^−1^ organic C, 0.93 g kg^−1^ total N, 9.57 mg kg^−1^ Olsen P, 191 mg kg^−1^ exchangeable K, 92.5 g kg^−1^ CaCO_3_ and had a pH of 8.62 across all plots, with little inter-plot variation (as previously reported by Yang *et al*.^25^). The experimental site has a mean annual temperature of 13.0 °C and mean annual precipitation of ca. 550 mm, which falls mainly from June to September.

The field experiment was laid out with a series of 196 m^2^ (14 m by 14 m) plots which included three soil management regimes. The first of these was bare fallow, with no growing vegetation, fertilizer or manure inputs, and weeds manually controlled (hoed); it was plowed annually during October (hereafter referred to as Fallow). The second regime consisted of abandonment, with no artificial perturbation, and allowing vegetation to grow naturally; this resulted in numerous species of herbaceous plants and a few arboreous individuals thriving (Abandon). The third was a winter wheat (*Triticum aestivum* L.)-summer maize (*Zea mays* L.) rotation system, with two crops per year (Cropping). Four nutrient management treatments were applied in conjunction with the Cropping regime: no fertilizer or manure inputs (control, hereafter CONTROL); combinations of inorganic N, P and K fertilizers (NPK), and NPK plus wheat straw or maize stalk (SNPK) or dairy manure (MNPK, where M refers to dairy manure). The NPK treatment received 165.0 kg N ha^−1^, 57.6 kg P ha^−1^ and 68.5 kg K ha^−1^ in the winter wheat season and 187.5 kg N ha^−1^, 24.6 kg P ha^−1^ and 77.8 kg K ha^−1^ in the summer maize season (June to September). The SNPK treatment received the same rates of N, P and K as the NPK treatment, with an additional 4500 kg ha^−1^ of wheat straw (air-dried) annually from 1990 to 1998, and all above-ground maize stalk from the plot; the latter had a mean annual weight of 4392 kg ha^−1^ (ranging from 2630 to 5990 kg ha^−1^) since 1999. The added straw/stalk was manually chopped into ca. 3 cm pieces and incorporated into the soil in autumn before sowing winter wheat. Dairy manure was also added once a year before sowing wheat. The MNPK treatment was given 1.5-fold as much N, inorganic P and K as the NPK treatment, but 70% of the N was from organic manure in the winter wheat season. In the summer maize season, the MNPK treatment received the same rates of N, P and K from inorganic fertilizers as in the NPK treatment without the addition of organic manure. The C and N contents of the manure were 26.45% ± 7.82 (SD) and 1.32% ± 0.91 respectively. The annual mean dry weight of organic manure applied in the MNPK treatment over the years of experimentation was 20.6 ± 10.8 tonnes. All inorganic fertilizers and organic materials applied were incorporated into the soil to the depth of plowing (ca. 15–20 cm) before winter wheat was sown and about one month after maize was planted. Nutrient forms were: N as urea, P as single superphosphate and K as potassium sulfate. Winter wheat was sown in October and harvested in the following June, then summer maize was planted and harvested about three months later, at the end of September or in early October. The plots were irrigated with groundwater 1 to 2 times during the winter wheat season, and 2 to 4 times during the summer maize season, depending on crop needs. Irrigation supplied approximately 90 mm of water on each occasion. All above-ground crop residues were removed after harvest unless otherwise specified. The fields were conventionally tilled.

### Soil sampling and analysis

Undisturbed soil samples were collected at the beginning of June 2011, one week before the winter wheat harvest. For each treatment, three replicate composite samples were taken, with each composite consisting of three undisturbed soil cores (10 cm in diameter and 10 cm in height). The sampling depths were 0–10, 10–20 and 20–30 cm. Field-moist soil was gently broken apart along natural break points and passed through a 10 mm sieve. Plant and organic debris in the sieved soil were carefully removed with forceps, and the soil samples were then air-dried. Subsamples of 200 g were then shaken through a motorized sieving device with opening diameters of 2, 1, 0.5 and 0.25 mm for 5 minutes to obtain five size fractions: >2, 2–1, 1–0.5, 0.5–0.25 and <0.25 mm. The soil retained by each sieve was weighed. From each dry sieving fraction, subsamples of 100 g were used to get water stable aggregates using the wet sieving technique (Yoder, 1936)^44^. In this procedure, 50 g of dry-sieving aggregates were placed on the topmost of a nest of sieves of diameters 2, 1, 0.50, 0.25 and 0.053 mm, and pre-soaked in water for 5 min before shaking in water at 30 oscillations per minute (with an amplitude of 3.5 cm) for three minutes. For each sample this procedure was performed twice, using 100 g per sample in total. The resultant aggregates on each sieve were oven-dried at 50 °C for 48 h before their masses were recorded. The mass of the <0.053 mm fraction was obtained by the difference between the initial mass and the mass of soil retained on all the sieves. The percentages of water stable aggregates were determined and mean weight diameter (MWD) was calculated as:


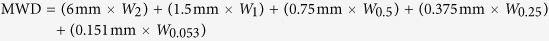


where *W* is the proportion of aggregates in each size class.

Bulk soil was analyzed for soil properties and aggregating agents. Soil pH was measured in a 1:2.5 soil: water extract by pH meter. Electric conductivity (EC) was measured in a 1:5 soil: water extract by EC meter. SOC was determined by potassium dichromate (K_2_Cr_2_O_7_) oxidation at 170–180 °C followed by titration with 0.1 mol L^−1^ ferrous sulfate^45^. Total N was determined by the Kjeldahl method after H_2_SO_4_ digestion in the presence of K_2_SO_4_-CuSO_4_-Se as catalyst^46^. Olsen P was measured by extracting the soil sample with 0.5 mol L^−1^ NaHCO_3_ (pH 8.5), after which the phosphorus concentration of the extract was determined using the procedure of Li^47^. Soil exchangeable K was extracted using 1 mol L^−1^ ammonium acetate (pH 7.0) and measured using a flame photometer^47^. Soil particle size distribution was analyzed by the pipette method and bulk density by the core method^47^.

We measured the length of hyphae in a 4 g subsample of soil using an aqueous extraction/filtration technique followed by microscopic quantification of hyphae at 200X magnification^48^. Components of soil organic matter, i.e. pentose, hexose, humic acid and fulvic acid, were determined using procedures described by Wen (1984)^45^. Free iron and aluminum oxides in soil were analyzed following the method of Jackson *et al*.^49^. CaCO_3_ was determined following the method of Bundy and Bremner^50^.

Data analysis. For each variable, a mean value was obtained from the results for the three composite samples, and significant differences between the means were identified by performing one-way analysis of variance; the LSD was computed to compare the means of above variables (*P* < 0.05). Two-way analysis of variance was also applied to check the effects of treatment and depth on the tested variables and their interactions.

## Additional Information

**How to cite this article**: Zhang, S. *et al*. Soil aggregation and aggregating agents as affected by long term contrasting management of an Anthrosol. *Sci. Rep.* **6**, 39107; doi: 10.1038/srep39107 (2016).

**Publisher's note:** Springer Nature remains neutral with regard to jurisdictional claims in published maps and institutional affiliations.

## Figures and Tables

**Table 1:** Soil chemical and physical properties after 21 years of the experiment in Shaanxi Province, China and two-way ANOVA test of the effects of treatment and depth and their interaction on soil properties, as shown by *P* values.

Treatment†	SOC (g/kg)	TN (g/kg)	Olsen P (mg/kg)	Available K (mg/kg)	pH	EC (μs/cm)	CaCO_3_ (g/kg)	Clay (%)
**0–10 cm**								
Abandon	14.3 A‡	1.48 A	9.50 A	336 A	8.3 A	139 A	75.6 A	35.1 A
Fallow	9.02 B	1.05 B	7.12 A	245 B	8.3 A	121 AB	80.6 A	33.4 A
CK	8.37 Bd	1.00 Bd	2.25 Bd	132 Cc	8.3 Aa	107 Bc	80.5 Aa	33.1 Aa
NPK	11.7 c	1.40 c	43.8 c	407 b	8.3 a	142 b	70.9 a	33.8 a
SNPK	14.1 b	1.54 b	69.9 b	445 b	8.2 b	148 b	78.4 a	32.4 a
MNPK	20.9 a	2.05 a	288 a	509 a	8.1 c	210 a	75.6 a	36.2 a
**10–20 cm**								
Abandon	8.23 A	0.68 B	1.65 A	150 A	8.3 A	104 A	86.7 A	32.0 A
Fallow	8.00 A	0.67 B	1.14 A	115 B	8.3 A	111 A	87.9 A	33.9 A
CK	7.92 cB	0.85 dA	1.39 cA	101 cB	8.4 aA	107 cA	82.9 aA	34.4 abA
NPK	10.4 b	1.11 b	25.8 b	198 b	8.4 a	147 b	77.6 a	36.9 a
SNPK	11.0 b	0.93 c	19.1 b	219 ab	8.3 a	146 b	79.0 a	32.0 b
MNPK	15.6 a	1.44 a	125 a	269 a	8.3 a	202 a	86.6 a	35.8 a
**20–30 cm**								
Abandon	6.85 A	0.51 B	2.12 A	101 A	8.4 A	95 A	83.9 A	—
Fallow	7.12 A	0.42 C	1.68 A	91 B	8.4 A	94 A	81.3 A	—
CK	6.86 bA	0.65 bA	0.73 cB	89 bB	8.5 aA	106 cA	76.6 aA	—
NPK	7.87 b	0.73 a	9.63 b	102 b	8.5 a	125 ab	82.2 a	—
SNPK	7.84 b	0.74 a	8.97 b	102 b	8.5 a	122 b	87.0 a	—
MNPK	9.75 a	0.62 b	25.3 a	132 a	8.3 a	138 a	98.2 a	—
***P*** **values**								
SM	0.000	0.000	0.000	0.000	0.222	0.375	0.515	0.975
Depth (D)	0.000	0.000	0.000	0.000	0.014	0.000	0.055	0.590
SM × D	0.000	0.000	0.000	0.000	0.402	0.005	0.489	0.072
F	0.000	0.000	0.000	0.000	0.030	0.000	0.116	0.002
D	0.000	0.000	0.042	0.000	0.003	0.000	0.032	0.148
F × D	0.000	0.000	0.001	0.000	0.245	0.000	0.049	0.157

^†^CK, no fertilizer; NPK, mineral fertilizers; SNPK, combination of crop straw (S) and NPK; MNPK, combination of dairy manure (M) and NPK; SM, soil management regimes; F, fertilization treatments.

^‡^Different capital letters within each column indicate significant differences between soil management treatments (Abandon, Fallow and CK) at 0.05 (n = 3); different small letters indicate significant differences between fertilization treatments (CK, NPK, SNPK and MNPK) under the cropping system at 0.05 (n = 3).

**Table 2:** Distribution of water stable aggregates (%) and mean weight diameter (MWD) under different long term soil management regimes.

Treatment	Aggregate distribution (%)					MWD (mm)
	>2 (mm)	2–1 (mm)	1–0.5 (mm)	0.5–0.25 (mm)	0.25–0.053 (mm)	
**0–10 cm**						
Abandon	52.9 A	17.1 A	9.8 B	6.3 B	9.8 B	3.70 A
Fallow	23.1 C	17.6 A	16.2 A	16.0 A	21.4 A	1.98 B
CK	33.7 Ba	13.6 Ac	13.1 ABb	13.6 Aa	22.1 Aa	2.50 Ba
NPK	22.4 b	19.8 b	21.7 a	17.3 a	15.7 bc	1.95 a
SNPK	27.4 ab	26.2 a	20.7 a	12.6 a	11.5 c	2.29 a
MNPK	12.5 c	23.1 ab	24.6 a	18.9 a	17.7 ab	1.42 a
**10–20 cm**						
Abandon	36.8 A	20.0 A	13.5 A	10.9 B	14.8 B	2.78 A
Fallow	18.5 B	17.3 A	16.1 A	16.0 A	26.5 A	1.68 B
CK	38.8 Aa	17.2 Ac	12.5 Aa	10.2 Bb	18.8 Bab	2.82 Aa
NPK	32.3 a	23.5 ab	14.3 a	11.8 ab	14.9 bc	2.54 a
SNPK	37.8 a	19.8 bc	16.0 a	11.4 ab	12.3 c	2.81 a
MNPK	15.2 b	24.2 a	17.7 a	15.1 a	23.5 a	1.57 a
**20–30 cm**						
Abandon	28.9 A	19.5 A	13.7 A	12.2 B	20.4 C	2.31 A
Fallow	10.4 B	10.9 C	17.1 A	20.2 A	36.1 A	1.10 B
CK	25.4 Aa	15.1 Ba	14.3 Aa	13.2 Bb	27.8 Ba	2.03 Aa
NPK	16.1 ab	16.0 a	17.1 a	17.0 ab	29.6 a	1.50 a
SNPK	14.2 b	15.9 a	22.7 a	17.5 a	23.6 a	1.45 a
MNPK	6.8 c	14.1 a	21.9 a	20.4 a	30.4 a	0.97 a
***P*** **values**						
SM	0.000	0.039	0.005	0.000	0.000	0.000
Depth	0.000	0.139	0.228	0.003	0.000	0.006
SM × D	0.040	0.003	0.582	0.003	0.025	0.375
F	0.000	0.009	0.000	0.000	0.000	0.003
Depth (D)	0.000	0.000	0.002	0.000	0.000	0.002
F × D	0.101	0.037	0.338	0.332	0.198	0.944

The abbreviations used for treatments and statistical presentation are the same as in Table 1 (n = 3).

**Table 3:** Pearson bivariate correlation coefficients between MWD, proportions of aggregates and aggregating agents and soil properties under three soil management regimes (CK, Fallow and Abandonment) (n = 9).

	MWD	Water stable aggregates (mm)				
		>2	2–1	1–0.5	0.5–0.25	0.25–0.053
MWD	1	0.998^**^	0.500	−0.962^**^	−0.971^**^	−0.945^**^
HL	0.841^**^	0.865^**^	0.148	−0.886^**^	−0.819^**^	−0.669^*^
Pentose	−0.399	−0.419	−0.038	0.432	0.458	0.246
Hexose	0.582	0.576	0.077	−0.537	−0.480	−0.568
HA	0.618^*^	0.629^*^	0.031	−0.573	−0.504	−0.549
FA	0.865^**^	0.888^**^	0.148	−0.912^**^	−0.854^**^	−0.682^*^
CaCO_3_	−0.299	−0.326	0.402	0.401	0.272	0.097
Free Fe oxide	−0.344	−0.380	0.537	0.446	0.202	0.154
Free Al oxide	0.640^*^	0.676^*^	−0.128	−0.751^**^	−0.651^*^	−0.388
SOC	0.702^*^	0.698^*^	0.155	−0.650^*^	−0.609^*^	−0.674^*^
TN	0.665^*^	0.669^*^	0.014	−0.630^*^	−0.532	−0.615^*^
Olsen P	0.515	0.499	0.159	−0.417	−0.400	−0.564
AK	0.608^*^	0.591^*^	0.245	−0.489	−0.494	−0.664^*^
EC	0.590^*^	0.577	0.218	−0.482	−0.509	−0.607^*^
pH	−0.439	−0.416	−0.323	0.282	0.277	0.589^*^

MWD, mean weight diameter; HL, hyphal length; HA, humic acid; FA, fulvic acid; SOC, soil organic carbon; TN, total nitrogen; AK, K extracted with 1 mol NH_4_OAC; EC, electrical conductivity. ** and * indicate correlations significant at the 0.01 and 0.05 level respectively (1-tailed).

**Table 4:** Pearson bivariate correlation coefficients between MWD, proportions of aggregates and aggregating agents and soil properties for treatments under Cropping (n = 12).

	MWD	Water stable aggregates (mm)				
		>2	2−1	1−0.5	0.5−0.25	0.25−0.053
MWD	1	0.996^**^	0.154	−0.718^**^	−0.932^**^	−0.651^**^
HL	0.618^*^	0.659^**^	−0.416	−0.583^*^	−0.591^*^	−0.088
Pentose	−0.079	−0.157	0.848^**^	0.434	0.085	−0.522^*^
Hexose	−0.254	−0.315	0.612^*^	0.582^*^	0.299	−0.352
HA	−0.359	−0.415	0.567^*^	0.497	0.377	−0.182
FA	−0.282	−0.356	0.811^**^	0.503^*^	0.227	−0.329
CaCO_3_	−0.497	−0.464	−0.379	0.117	0.361	0.601^*^
Free Fe oxide	−0.191	−0.169	−0.230	−0.079	−0.022	0.371
Free Al oxide	0.162	0.174	−0.059	−0.082	−0.081	−0.073
SOC	−0.240	−0.312	0.746^*^	0.566^*^	0.275	−0.413
TN	−0.103	−0.178	0.764^*^	0.509^*^	0.189	−0.535^*^
Olsen P	−0.347	−0.407	0.565^*^	0.641^*^	0.444	−0.290
AK	−0.076	−0.152	0.763^**^	0.595^*^	0.192	−0.621^*^
EC	−0.328	−0.401	0.747^**^	0.545^*^	0.332	−0.319
pH	0.020	0.087	−0.679^*^	−0.463	−0.124	0.572^*^
Clay	−0.481	−0.485	0.328	0.098	0.320	0.392

MWD, mean weight diameter; HL, hyphal length; HA, humic acid; FA, fulvic acid; SOC, soil organic carbon; TN, total nitrogen; AK, K extracted with 1 mol NH_4_OAC; EC, electrical conductivity. ** and * indicate correlations significant at the 0.01 and 0.05 level respectively (1-tailed).
